# Exploring how different modes of governance act across health system levels to influence primary healthcare facility managers’ use of information in decision-making: experience from Cape Town, South Africa

**DOI:** 10.1186/s12939-017-0660-5

**Published:** 2017-09-15

**Authors:** Vera Scott, Lucy Gilson

**Affiliations:** 1 School of Public Health, University of the Western Cape, Cape Town, South Africa; 2 Health Policy and Systems Division School of Public Health and Family Medicine, University of Cape Town, Cape Town, South Africa; 3 Health Economics and Systems Analysis Group, Department of Global Health and Development, London School of Hygiene and Tropical Medicine, London, UK

## Abstract

**Background:**

Governance, which includes decision-making at all levels of the health system, and information have been identified as key, interacting levers of health system strengthening. However there is an extensive literature detailing the challenges of supporting health managers to use formal information from health information systems (HISs) in their decision-making. While health information needs differ across levels of the health system there has been surprisingly little empirical work considering what information is actually used by primary healthcare facility managers in managing, and making decisions about, service delivery. This paper, therefore, specifically examines experience from Cape Town, South Africa, asking the question: How is primary healthcare facility managers’ use of information for decision-making influenced by governance across levels of the health system? The research is novel in that it both explores what information these facility managers actually use in decision-making, and considers how wider governance processes influence this information use.

**Methods:**

An academic researcher and four facility managers worked as co-researchers in a multi-case study in which three areas of management were served as the cases. There were iterative cycles of data collection and collaborative analysis with individual and peer reflective learning over a period of three years.

**Results:**

Central governance shaped what information and knowledge was valued – and, therefore, generated and used at lower system levels. The central level valued formal health information generated in the district-based HIS which therefore attracted management attention across the levels of the health system in terms of design, funding and implementation. This information was useful in the top-down practices of planning and management of the public health system. However, in facilities at the frontline of service delivery, there was a strong requirement for local, disaggregated information and experiential knowledge to make locally-appropriate and responsive decisions, and to perform the people management tasks required. Despite central level influences, modes of governance operating at the subdistrict level had influence over what information was valued, generated and used locally.

**Conclusions:**

Strengthening local level managers’ ability to create enabling environments is an important leverage point in supporting informed local decision-making, and, in turn, translating national policies and priorities, including equity goals, into appropriate service delivery practices.

## Background

Governance and information have been identified as key, interacting levers of health system strengthening [1]. Governance is also increasingly understood as a systems-level phenomenon: entailing decision-making processes [2, 3] that influence performance and that are spread across the macro (global and national), meso (organisational and local) and micro (individual interactions) levels of the health system [4, 5]. In other words, governance is about ‘solving problems and creating opportunities, and creating the structures and processes for doing so’ [6] p69. Health information, meanwhile, is commonly understood as a tool of governance, used in decision-making and allowing oversight of resources deployed and outcomes achieved [7]. There has, therefore, been considerable focus on and investment in health information systems (HISs) for health system strengthening in low and middle income countries [8–11].

An extensive literature has detailed the challenges of supporting health managers to use formal information from the HIS in their decision-making [12–17]. However, whilst it is recognised that health information needs differ across levels of the health system [9, 18], there has been surprisingly little empirical work considering what information is actually used by primary healthcare (PHC) facility managers in managing, and making decisions about, service delivery. Yet these managers play critical roles in strengthening health systems [19]. Managing at the coalface of implementation, they also influence the translation of policy intentions and national priorities – including equity goals -into health service delivery and patient/community experiences [20]. For example, with specific reference to the critical health policy goal of equity, these facility managers influence which, if any, clients are prioritized for treatment inside the facility and the extent and focus of outreach activities beyond the facility walls. Although equity was not a specific focus of the research reported in this paper, the findings are helpful in thinking about how to support local-level action towards equity goals.

This paper, therefore, specifically examines experience from Cape Town, South Africa, asking the question: How is primary healthcare facility managers’ use of information for decision-making influenced by governance across levels of the health system? The research is novel in that it both explores what information these facility managers actually use in decision-making, and considers how wider governance processes influence this information use – adding to the small body of relevant, empirical work [21–23]. Ultimately, the paper shows how higher level governance processes shape what information and knowledge is valued – and, therefore, used at lower system levels. It offers insights of relevance to those responsible for national health policy design and governance and those working at sub-national level to support health managers in the public sector, as well as to the wider global health research communities interested in understanding how PHC facility managers can be supported to improve health outcomes in Low and Middle Income Countries (LMICs).

## Methods

This research is nested within a larger project - the District Innovation, Action and Learning for Health System Development (DIALHS) project - which has been described in detail elsewhere [24–26]. Located in the emerging field of Health Policy and System Research*,* the research draws on constructivist and participatory perspectives, in particular the notion that reality is not just socially constructed but co-constructed, and that participation is intrinsically of value.

The research site is Mitchells Plain, Cape Town. The geographical area has a population of just over 900,000 residents with 29% of the population living in informal settlements and high levels of unemployment (32% adults aged 15 to 64) and poverty (61% households with monthly income of R3 200 or less). There is a quadruple burden of disease with significant mortality from human immunodeficiency virus (HIV), other infectious diseases, non-communicable diseases and injuries [27, 28]. The top ten causes of death include: homicide, human immunodeficiency virus, tuberculosis, lower respiratory infections, road traffic accidents, diabetes mellitus, ischaemic heart disease, low birth weight and stroke. Public health service delivery in Mitchell’s Plain currently (2016) falls under the dual authority of the Metro District Health System (MDHS) of the Western Cape Department of Health (provincial government) and City Health, the health department of the City of Cape Town (local government). There are three types of public primary healthcare services which vary in services offered and size of staff complement. Broadly, there are 8 clinics which have between 8 and 20 staff members and provide at least basic preventative services (such as family planning and HIV testing) and child curative care for common childhood illnesses. The 6 community day centres have between 24 and 68 staff members and offer predominantly general adult curative services. The 3 larger community health centres have between 143 and 180 staff members; in addition to adult curative services they also have 24 h emergency and obstetric units.

Four facility managers (one from MDHS and three from City Health, selected because they represented facilities of different sizes and the two organisations), and the first author (VS) were co-researchers in a multi-case study which involved cycles of data collection and collaborative analysis with individual and peer reflective learning over a period of three years. Three decision-making areas of management were chosen as cases to provide an insight into how the health system works at the point of primary healthcare implementation. These cases were also specifically identified by subdistrict managers as vital to facility and health system performance, having the potential to act as levers of local health system strengthening. The cases were: improving efficiency of service delivery, implementing programme priorities and managing leave of absence by staff. The findings from the MDHS and City Health facility experiences are largely presented together in this paper as they were similar in nature; where differences exist they are noted.

**Fig. 1:**
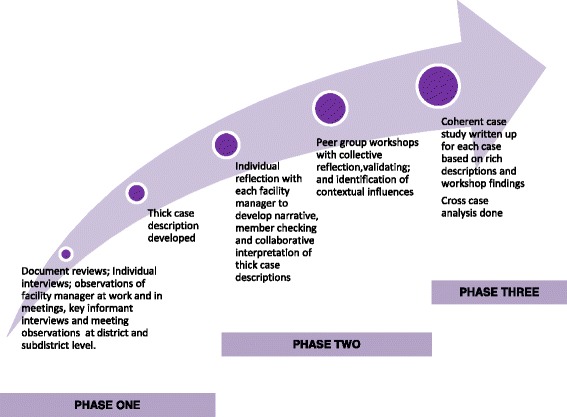
Phases of data collection and analysis in this multi case study

Data collection and analysis was undertaken over three phases as shown in Fig. 1., with each phasing taking approximately one year. In the first phase a document review of national, provincial and district policy and guidelines pertaining to the three cases was conducted to understand the policy context and the intended approach to management of each case. This was followed by participant observation of the four facility managers at work, both in their facilities and in sub district management meetings (32 observations totalling 80.5 h). Next a set of in-depth interviews was conducted with the facility managers using story telling techniques and mind maps (21 interviews lasting between 45 min and two hours five minutes). In parallel a set of 31 key informants (district and sub-district managers and support staff working in health information, finance, human resources and programmes) were interviewed in order to understand the processes, values and attitudes operating in the sub district and district context, and district meetings were observed. These data were collated into a rich description for each case. In the second phase the 4 facility managers worked with each rich description and engaged in a deliberate process of individual reflective learning which added to the data and to the interpretation of the three emerging narratives. These narratives then informed two sets of three workshops, one set with each of the two teams of facility managers in the sub district (City Health and MDHS), involving a total of 20 facility managers (over the course of the 3 years there was some change in facility managers; new appointees were included in the workshop series). The workshops enabled peer validation of findings and testing of generalizability, as well as cycles of collective reflective learning and collaborative analysis in each peer group on each case. In the third phase the case studies were written up based on the individual narratives and peer group workshops and a cross case analysis undertaken. Multiple strategies to strengthen rigour were employed throughout, including having a prolonged period of engagement [29]; creating an audit trail of evidence and steps in interpretation [30]; triangulation of data from multiple methods and multiple sources [30] to “develop a complex picture of the phenomenon being studied” [31]. Peer debriefing and review [29] in monthly operational and bi-annual reflective meetings of the DIALHS project team allowed emerging analyses and interpretations to be tested in the light of a broader suite of project work, dealing with health system governance and relationships between actors (examples of this other work can be found in [25, 26, 32]).

### Theoretical underpinnings

We have drawn on Hill and Hupe’s analytical framework of governance [33] in this paper, as it offers particular insights into the practice of governance as decision-making at the implementation or front line level of public systems. The framework adopts a systems (or multi-level) perspective on governance, and recognises governance is concerned with processes as well as structures. These authors suggest that it is important to explore *how* governance is enacted as this has consequences for the forms of relationships set up within and between levels of a public system, and for the forms of accountability established. They refer to the *how* of governance as the *modes of governance*. Drawing on work by Lindblom who described three mechanisms of social control [34] (in Hill and Hupe, 2007), the authors propose three modes of governance - authoritarian, transactional and persuasion - which are linked to forms of accountability. They understand accountability to be a “social relationship in which an actor feels an obligation to explain and to justify his conduct to some significant other” [35] in Hupe&Hill, 2007, p286; the essence of accountability is therefore answerability [36], having an obligation to “give account” of decisions or actions.

The authoritarian mode of governance operates along the lines of the more traditional understanding of public bureaucracies, in which the centre exercises power over the system by demanding compliance to rules and standard operating procedures. The nature of accountability is one of task enforcement within a hierarchical structure of relationships. At the level of implementation Hill and Hupe call this the enforcement mode. In the transactional mode (developed from the idea of market exchange), power is exercised through contractual arrangements: performance frameworks are created and targets are set which then shape subsequent performance. This approach has been promoted as part of the New Public Management approaches [37] that emerged in the late 1980s, which give managers greater discretion in decision-making and use incentive-based motivation towards targets. In the transactional mode accountability remains vertical across levels of the health system, but is exercised through contractual relationships. It is target-oriented, seeking to promote performance; it values indicators and an assessment of outputs. At the level of implementation Hill and Hupe call this the performance mode. In governance by persuasion, the central action is to give direction by creating a shared vision, and by then ensuring an environment that enables others, including those lower in the system, to exercise discretion and to participate in co-producing the path to that vision. There is a more horizontal form of accountability within and between levels around the agreement to work towards shared goals and internalised professional standards. This mode involves relationships of trust and is described as being more orientated to achieving the desired impact, rather than particular outcomes. At the level of implementation Hill and Hupe call this the co-production mode.

Hill and Hupe argue that in practice governance entails choices about ways of governing, often made simultaneously at several layers within a policy process or within a system. Several modes of governance may, thus, be operational within a given setting.

## Results

This section starts by exploring how a range of different forms of information and knowledge were used (and were required) in the decision-making world of the PHC facility managers. From this starting point it then traces how the subdistrict modes of governance influenced what information and knowledge was valued. It then shifts focus to the broader health system context to examine the influence of national, provincial and district managerial processes and values over the subdistrict and PHC facility level. Finally, drawing on the empirical findings, it posits a model of how modes of governance work across levels of the health system to influence the use of information in facility level decision-making, and discusses the implications of this for supporting local informed decision-making and enabling local health system strengthening.

### Different forms of information and knowledge in decision-making at facility level

In managing the coverage of health services and priority programmes, facility managers worked with routine health information from the HIS and supplemented this with clinical audits to assess the quality of care. Table 1 illustrates how a facility manager used routine information to identify a problem (low HIV testing in children under 5 years) and, having followed this up with an ad hoc clinical audit to assess if the Integrated Management of Childhood Illnesses (IMCI) protocols were being followed, she used the routine data to plan, monitor and encourage her staff to improve the diagnosis of HIV in children. Some elements of the routine HIS were more useful in longer-term monitoring and planning. For example, monthly headcounts attracted more managerial interest in a quarterly review assessing accessibility. Combined with a three-yearly district-wide waiting time survey they were used to inform process engineering of patient flow within facilities to improve service delivery efficiency.

**Table 1:** Using information from the RHIS, complemented with clinical audit data

Facility manager 1, interview 12 Aug 2012
*What I saw within my folder audits, I noticed that there was a trend between the PNs (professional nurses) and now I wasn’t sure whether it was because they were just lazy to do the HIV test, because on average every staff member needs to do at least 3 per month (to reach our targets). If you tick off (on the pre-designed IMCI consultation sheet) one of the problems a child comes in with, it could be growth faltering, it could be pneumonia, any one of those, oral thrush, then they are writing there ‘HIV unlikely’ whereas the IMCI diagnosis should be ‘possible HIV infection’, and that should prompt the nurse to offer the mom and the child an HIV test. And in following up, we actually found 2 children who were HIV positive and that to me was very heart sore, because the one child was coming to the facility regularly and that child was growth faltering, but nobody picked it up.*
*I actually presented the case at the subdistrict, because just to make the other managers aware as well, because if the PN’s in my facility are doing it, ten to one they doing it in the other facilities. And when we had a discussion on it and then after the other facility managers said that they are seeing the same. And then I spoke to my staff, I told them this is the problem and I’m very heart sore because these children should have been on ARVs ages ago and then we decided together…. this can be a quality improvement (project) number 1, plus this is important, so this is one of my pet projects that I took on….We started this about 4 months ago so it’s fairly new and I’m monitoring it; the numbers are increasing in the children under 5, that they are testing…*
*When I go into the staff room, I always compliment them, when they’re sitting there, “Ooh! The HIV test (monthly statistics), excellent!” And I tell them also at the end of the month; do you know how much we did? We did 84. Last month I told them and Sister X was actually the one that was saying ‘Good sister, excellent, you see we are actually working’, so I said to her, ‘I’m not saying you guys aren’t working, you are and you doing good work, you know?’*

In contrast the routine health management information in the human resource and procurement information systems was not reliable enough to be used for local decision-making as leave application forms were often lost in transfer to the subdistrict office where they should have been captured in the institutional database, or were not captured on time (backlogs of a few months were not unusual). To identify staff members who were exhausting their sick leave through frequent unplanned leave, facility managers developed their own parallel reporting systems e.g. an Excel spreadsheet to track leave usage; or an individual staff profile of leave mapped onto a monthly calendar to identify patterns suggestive of abuse (such as unplanned leave clustered around weekends and public holidays). These innovations show a high level of commitment to generating and using formal information in the face of an inadequate human resource information system.

Informal information was also found to be important in facility level decision-making across the three cases. Facility managers regarded the information gathered through regular walking rounds of their facilities as invaluable: being able to see for themselves what was happening in areas of service delivery as well as ‘behind the scenes’; being able to engage with staff along a continuum which could be characterised at one end as “requesting a verbal report” and the other end as “having a quick chat”.

*(Doing walking rounds) there are people stopping you, they are asking questions, there are things that you are noticing: the BP machine is not working…you were not actually doing the equipment audit but you end up doing it because you see now there are long queues because people can’t actually get their blood pressures done because there is only one blood pressure machine working instead of four. And the other three are standing there not functioning but the staff did not send them for repairs. You receive information from the staff that are actually working in those points.*

Workshop 4 November 2013.

Facility managers were also not only users of information, but also played a key role in *generating* the various types of information and knowledge that they needed to make decisions. Informal information, in the form of observational data, reports from staff and client complaints information was more useful for immediate problem-solving on days when facilities were congested. In deciding how to reallocate workload and streamline processes on busy days, or when the facility was short-staffed, facility managers also used what they knew about particular staff members, who coped with what level of stress and how teams could be configured to work most efficiently.

*Do I take a person and put a person who can actually do two things at the same time, or what do I do? So these are some of the questions that come to mind as you are allocating people. But these are the things that are not written anywhere… you actually have to think about them on the spot when you are doing the allocation.*

Workshop 4 November 2013.

Similarly in managing absenteeism, some of the facility managers drew extensively on highly particular knowledge of staff members, their personal and family circumstances, their patterns of unplanned leave and what factors could be used to motivate better attendance. The policy guidelines for managing leave created the space for facility managers to institute corrective rather than punitive measures to address absenteeism, and there were examples of interventions that were highly individualised. Informal information in the form of verbal reports from staff members, overheard conversations, impressions and hunches, though not measurable, were seen as valuable in assessing levels of staff morale at facility level. The devolution of human resource management responsibilities from the district to PHC facilities was new during the time of this research, and managers were experimenting with how to keep records of meetings with staff members to document and formalise some of this information.

Another form of knowledge which featured prominently in decision-making was knowledge gained from the experience of having managed the same or a similar problem before, and having learnt from that experience. Many of the challenges that facility managers faced across the three cases recurred repeatedly over time, such as: being short-staffed; having a staff member who abused unplanned leave; having congested service points with longer than average waiting times which delayed the smooth flow of clients through the facility; recording statistics which showed a low coverage of a priority health programme service. When asked what informed a particular decision, facility managers often prefaced their response with phrases such as “*last time this happened*” or “*what I have already learnt*”. This experiential knowledge was evident in interviews with individuals and, importantly within the health system context, there was evidence that it could also be generated and owned by a collective, as illustrated in the case vignette of an immunisation campaign described in Table 2. When the national department of health decided to run a Pneumococcal Conjugate Vaccine immunisation targeted at children 18–35 months old for the first time in 2012, the subdistrict managers anticipated that the strategies required to reach such a narrow age target would be different from those used to reach children under 5 in the more familiar measles immunisation campaigns. They decided to set up a dedicated meeting for facility managers and their immunisation teams to share experiences, and to generate local experiential knowledge on how to reach children in this target age group.

**Table 2:** Experiential knowledge being generated within the context of a novel immunisation campaign

The campaign initially focused on primary healthcare facilities and crèches but, on reviewing the statistics for the first two months, they realised that the yield here was far below the targets set. They suspected that, given the informal and oft times erratic nature of crèches in poorer areas, their list was incomplete so the task team decided to seek out other community sites to access children for vaccination. In April they decided to set up an immunisation station at local shopping mall, anticipating that mothers and their children would be found their after the pay-out of social grants or wages. While well-frequented, the space they were given in the mall was not in the public eye. They tried to ‘market their product’ by having community volunteers wearing orange bibs wander through the mall to advertise the campaign. The management of the mall objected to this so they then put up posters on boards in the foyer but found that even on a good day they would only get about 25 children to immunise. They tried various strategies such as going into shops and identifying children potentially in the right age but none were very successful. In their May meeting they decided to return to the communities with particularly low coverage and drive through the streets with a loudhailer. They found that many children in the target age group were at home with their mothers. The social grant pay-out queues and the community-based soup kitchens are also good to target. In their June meeting they discussed the importance of remaining flexible and trying different strategies in quick succession to find one that worked. From their experience they also learnt to anticipate that the venue of crèches and community soup kitchens would change over time, and to anticipate this next time they planned an outreach activity. They were surprised to find teenage mothers still in their pyjamas at 10 am in the morning and considered the implications of this for targeting other priority services such as family planning for under 18 year olds.	

### Subdistrict modes of governance influence what information and knowledge was valued

Different governance modes were observed within the subdistrict setting, each of which valued, generated and used different types of information. A strong emphasis in the subdistrict, present in both organisations and responding to the directives of the district and provincial offices, was placed on managing performance towards meeting targets, suggesting a transactional performance oriented mode. This placed high value on reliable and timeous routine information – the routine health information had to be reported to the subdistrict office by the 7th day of each month. The subdistrict office was then responsible for giving feedback to PHC facilities in the form of a data quality report to show data timeliness, completeness and accuracy, as well as a report on service delivery indicators. A standard operating procedure guided this process, designed to generate quality information to be used within a monthly review and planning cycle. During the time of this research, a monthly meeting solely on data quality was instituted in one of the organisations (in addition to the monthly management meeting described above), demonstrating the level of the subdistrict managerial investment in generating the quality of information required. Facility managers spent considerable time validating the data in their facilities and developed a number of parallel data collection systems, encouraged by subdistrict management, to allow cross checking of data variables, even drawing folders to check against the clinical notes when they found discrepancies.

*Because we are stats orientated, that is our big drive.*

Facility manager 1, interview 12 Aug 2012.

A review of key indicators from the routine HIS, disaggregated to facility level, was included in the monthly subdistrict facility management meeting. Facility managers were required to give account for their data quality and for their facilities’ performance against a set of facility targets. If a facility was falling behind in reaching their targets in priority programme areas, the facility manager was required to draw up an action plan to address this and this was reviewed in monthly supervisory visits to facilities (often only the presence of a plan rather than the content of a plan was assessed). Further, routine information was the basis of performance targets set within the individual target-based performance agreements facility managers signed with the subdistrict. They were held to account in a quarterly performance review meeting and, if they were not meeting their facility targets, then the contributing problems had to be identified and a plan of action developed as part of the individual manager’s workplace development plan.

Co-existing with the attention to information from the routine HIS, targets and performance management, an authoritarian mode of governance was observed in some of the key supervisory practices in the subdistrict. For example, there was a monthly supervisory visit to each facility, which was dominated by the administration of a detailed checklist quality assurance tool. Generating this information was valued in that it met the subdistrict’s requirements for compliance to the set of national core standards (described later). Both the transactional performance mode and the authoritarian mode of governance set up vertical accountability relationships requiring upward reporting of performance information and compliance checks.

While facility managers were committed to working with routine health information, they also experienced the subdistrict focus on upward reporting of information as limiting the support they received. The following quote, which refers to non-urgent clients being ‘deferred’- asked to return another day - on a regular basis (as a result of high service demand), illustrates how a problem not routinely measured was perceived to be invisible and of no concern to their subdistrict managers:

*Even the department, they don’t even want us to put those numbers down (of clients being deferred) for them to see. They just want us to give the headcount and the headcount doesn’t actually include the people that we are deferring. So in actual fact the senior managers don’t really want us to tell them about the people we are deferring… Sometimes I think for them it is what is put on paper versus what is really happening out there in the facilities.*

Workshop 4, November 2013 (MDHS 2).

PHC facility managers complained that they were called to an excessive number of ad hoc management meeting at the subdistrict level in order to give account of activities in their facilities. This reduced the time that they had to spend engaging with their staff and performing hands on management, and undermined their ability to access the informal information that they needed in their everyday problem-solving and management of staff. Instead the enforcement mode within the subdistrict supported the generation of formal written reports, verbal reports within meetings and quantifiable compliance measures such as those captured in the quality assurance tool.

A *co-production* mode of governance was, however, also evident at subdistrict level, in strategies that were being used to encourage collective learning and reflective practice. Subdistrict managers saw the value of experiential knowledge and, as illustrated in Table 1, sought to provide opportunities to support and generate such knowledge in their monthly management meetings with facility managers. This governance mode is also evident in the immunisation case vignette in Table 2: subdistrict managers deliberately created a space for learning from new experience. In this second example, the mode of co-production valued and used a wide range of information types: formal information in the form of campaign statistics were used to assess the effectiveness of outreach strategies, observations made by outreach team members, as well as their opinions, impressions and hunches were used to generate explanations for effectiveness. Experiential learning was then tested within group discussions and further experience in the field. Importantly, the co-production governance mode created horizontal relationships of peer accountability, with managers learning together and being accountable to one another in working towards common goals and objectives.

**Fig. 2:**
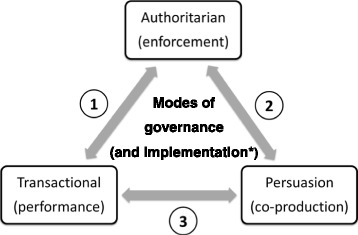
Possible shifts observed between the modes of governance. * the mode of governance corresponding to each mode of governance is shown in brackets.

Not only were all three governance modes evident in subdistrict management practices, but at times, also, the boundaries between the modes were blurred, as shown in Fig. 2. The modes of governance are shown in each block, alongside the linked accountability form at the implementation level. The following examples describe the three bi-directional arrows in Fig. 2, each representing a blurring between two modes of governance. The first arrow (1) shows a blurring between the performance and enforcement mode. While the use of targets in collective and individual performance management is typical of the performance mode, the way in which this was implemented in the subdistrict, through strong top-down planning processes, meant that it was experienced by facility managers as a control and command strategy (enforcement) with a requirement for upward reporting. Provincial targets were disaggregated first to districts and then to subdistricts. At subdistrict level, facility targets were given to facilities without any opportunity to give input into their appropriateness:

*(In the subdistrict planning) there was no opportunity to talk about targets. The only thing we had to plan around was services, things that they (the subdistrict) want, new programmes that they want. It’s like ‘How are we going to implement, what can we do?’ There was nothing about resources, targets, and things like that.*

Workshop 6 February 2014 (MDHS 3).

Facility managers questioned the feasibility of some of the targets which bore no resemblance to past performance; some were not contextually appropriate (e.g. targets for male medical circumcision in predominantly Muslim communities with high rates of infant circumcision). They reported that they and their staff became demotivated when it seemed impossible to reach targets. In contrast district and subdistrict managers reported with concern that there was “*no culture of information use*” among facility managers (District Plan-Do-Review meeting, June 2012) and sought to enforce further the use of the information from the routine HIS in facility level planning and monitoring, rather than negotiate around the targets.

The second arrow (2) shows a blurring between the enforcement and co-production mode. During the time of this research the subdistrict management in one organisation, working with the DIALHS team, experimented with different ways of structuring the monthly subdistrict management meeting with facility managers, recognising that it was not always effective in providing support to facility managers and rather was, at times, experienced as checking up and punitive (revealing an enforcement mode). Initially the purpose of the meeting was to give feedback on matters discussed at the district meeting, issue instructions and hold facility managers to account. The facility managers requested that the meeting should allow for more discussion and collective problem solving on the issues which they themselves placed on the agenda (a shift towards a co-production mode) and the subdistrict managers then introduced the opportunity for facility managers to take turns at sharing their experience of best-practice in dealing with commonly-encountered management problems, as referred to in Table 1.

The third arrow (3) shows a blurring between the performance and co-production mode. This is seen in the project meeting set up to support an immunisation campaign (Table 2) where different role-players were encouraged to set their own aspirational targets and come up with their own strategies (performance mode), but there was also a positive experience of collective reflective practice to generate new knowledge to support local innovation (co-production mode).

### Influence across levels of the health system: Managerial processes and values

**Fig. 3:**
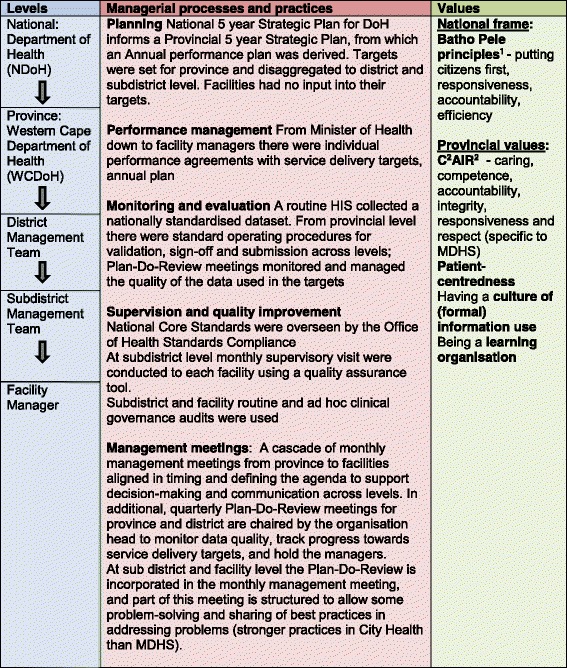
Governance operating through managerial processes and practices, and values, across the health system

The governance modes evident in the subdistrict context can, finally, be traced back to the influence of governance as exercised at higher levels of the health system, which operates through the managerial processes designed, and sets of values promoted. These are shown in Fig. 3. Key managerial processes include planning, performance management, monitoring and evaluation, and supervision.

The South African public health system, in common with many LMICs, functions as a bureaucracy with control exercised through a strong hierarchical structure [38]. Public sector policy, introduced by National Treasury [39–41], provides for a strong, coordinated approach to rational planning in all government departments and links planning and budgetary processes with performance management, which is seen as a tool to foster greater accountability, starting at the national level with the President and his ministers. In each department there are 5 year Strategic Plans (called National Service Delivery Agreements) and Annual Performance Plans. Targets, to express a “*specified level of performance that the institution, programme or individual is aiming to achieve within a given time*” [42] p11, are required at all levels of strategic and operational planning, and the progress towards meeting these targets has to be carefully monitored. Monitoring and evaluation has been strategically strengthened with the establishment of the Department of Monitoring and Evaluation (M&E) in the Presidency in January 2010, and by the development of a suite of policy frameworks from 2005 to 2011 supporting a government-wide monitoring and evaluation system [42–46]*.* Together with performance management systems, M&E systems promote vertical accountability relationships within the health system, with lower levels reporting up and being held accountable to higher levels. Within the National Department of Health (NDoH), the development of a HIS capable of supporting a district health system and providing performance information has been a priority in transforming the health system [47, 48]. While a range of components have been identified as part of a comprehensive HIS (including census, vital registration data and health resources records) it is the routine health service component which has been the first priority and which has attracted substantial managerial attention and resources [47]. Another key initiative to improve health service delivery has been the development of a set of National Core Standards, each with measurable criteria, to be overseen by the Office of Health Standards Compliance, a statutory body created in the National Health Act of 2003. A Provincial Inspectorate of Health Establishments was established to assess whether facilities met the norms and standards and oversee the accreditation of facilities. At a subdistrict level this process shaped the practice of supervisory visits to facilities in both organisations, which were focused on compliance and used checklists to generate audit information.

In line with national priorities, the City of Cape Town [49] and the Western Cape Department of Health (WCDoH) [50] adopted rational planning approaches and invested in improving the routine HIS. The WCDoH had a strong management structure, involving its district managers as part of the provincial management team which allowed seamless decision-making between provincial and district levels. The WCDoH also implemented a performance management system which was both collective and individual. Provincial targets were passed down the management ladder through a top-down process of annual planning and through the system of performance management, with service delivery targets being disaggregated from one level down to the next down to frontline staff in MDHS. City Health was responsible for meeting a proportion of these targets in specified services through a Service Level Agreement, and likewise disaggregated them and passed them down, but only to the level of the facility managers and not to facility staff. Province and the district (with MDHS and City Health both having an organisational head at district level) each held a quarterly monitoring and evaluation meeting called the Plan-Do-Review Meeting, respectively chaired by the provincial head of department and a district manager, to track progress towards the targets set in the planning processes, to manage data quality, and hold district and subdistrict managers to account. Table 3 illustrates the management practices within this meeting in more detail, showing how key indicators were reviewed and poor performing subdistricts were required to give account of their plans to reach their targets.

**Table 3:** Observation notes, District Plan-Do-Review meeting, June 2012

A set of over 20 indicators were reviewed systematically. Taking one indicator at a time the district performance since the start of the financial year was assessed against its target. If it was not meeting the target, then time was spend reviewing the performance against the pervious year’s performance for the quarter, and looking at trends over time. In additional any subdistrict that was not meeting the district performance average was asked to explain their situation and how they were addressing the problem.
In reviewing the couple year protection rate, the district manager pointed out that the district was generally not doing well. He highlighted the performance of (Subdistrict A) which had dropped below the baseline. He said that this indicator was driven by many component parts and that one priority component was contraception services to women under 18, and that this needed to be a particular focus going forward. The manager of Subdistrict A reported that she had done an analysis of this component but that this was now out of date and that she would repeat it. She said that this indicator was proving to be one of the most challenging. She said that her subdistrict strategy was one of service integration however, while this was successful in identifying new clients, they are not retaining these new clients in care. On monitoring performance of facilities over time they found that the statistics of some of the better performing facilities had dropped. She reported that further enquiry led them to find that staff didn’t really believe in strategy and weren’t committed to its implementation.
The district manager seemed satisfied with this report, and then invited the manager of Subdistrict B to explain her subdistrict’s poor performance in the couple year protection rate.

In this study the dominant mode of governance found to operate at the national level that also exerted influence down through the provincial and district levels of the health system to the subdistricts, was an authoritarian mode with top-down control and compliance to rules. This was supplemented by an orientation towards a transactional mode of governance through target-setting and performance management but this was also experienced by PHC facility managers as authoritarian, with enforced compliance.

Both governance modes were consistent with the national public sector Batho Pele^1^ principles which were developed in the early years post-apartheid to transform the public administration to be accountable and efficient, whilst also valuing being responsive and respectful of clients. However nearly two decades later the WCDoH recognised that its provincial value-base was still defined by “cost consciousness, bureaucracy, hierarchy, and confusing messages” [51], underpinned perhaps by the authoritarian mode of governance. The Department set out to redefine a set of visionary core values, aligned with the Batho Pele principles, in its vision for the year 2030 [51] captured in the acronym C^2^AIR^2^, seeking to demonstrate caring, competence, accountability, integrity, responsiveness and respect [52]. It also actively engaged in a wider change management process to foster the new values among managers and staff alike. Consistent with the dominant management values of vertical accountability and efficiency there were calls for “a greater culture of information” in district and subdistrict Plan-Do-Review meetings, which was understood as more use of formal quantifiable information in support of rational planning processes. However there was also a stated intention to promote a culture of learning and continuous improvement at all levels of the health system [51]. In discussion fora senior managers grappled with how to create an institutional environment that enabled experimentation and learning at district level (field notes, 2010 to 2015). Their intention to support this orientation created the space for subdistrict managers to introduce opportunities for peer learning into the routines of subdistrict practice, such as putting the sharing best-practice onto the agenda for monthly meetings.

Dominant modes of governance, supported by historicity, can have an inherent stability that is difficult to shift. An illustrative example of how difficult it was to challenge modes concerns an innovative attempt to change the nature of planning from an authoritarian top-down mode to a more inclusive bottom-up approach. In 2011 the subdistrict managers asked the DIALHS research team to support a one-day planning workshop. The intention was to develop a subdistrict plan which moved beyond a one-year planning cycle and which was informed by a highly inclusive community mapping exercise conducted earlier in May 2011 (described in Table 4). The planning workshop was well-attended and, encouraged to plan differently, facility teams explored new approaches to local action. The discussion of the problems identified in the community mapping exercise, and their contributing factors, was fruitful but teams struggled when they were asked to translate this understanding into concrete activities on planning templates as they found if difficult to merge the type of information (local context-specific tacit knowledge of communities) and priorities identified in the community mapping (a product of co-production) with the priority services represented in service delivery targets which used a different type of information (formal indicators from the HIS) generated in a different mode (the disaggregation from the provincial planning process being a product of enforcement and performance management). Importantly this attempt to plan differently failed because, despite an expressed intent on the part of the district to incorporate priorities identified by bottom-up planning, it was not possible challenge and modify the expectations set by higher level planning practices and outputs within the strict timelines they required, undermining the bottom-up process.

**Table 4:** Community mapping exercise, May 2011: eliciting local knowledge to inform local priority setting

A community mapping exercise, conducted in May 2011, was designed to help facility managers better understand the health needs of the communities they served, as well as appreciate the resources inherent in the community. A workshop with more than 80 participants brought subdistrict and facility managers together with representatives of civil society. Participants worked in area-specific groups defined by the primary healthcare facilities catchment areas. They were given large-scale maps of their area, and worked together to identify local health needs and to plot health resources and gaps onto their map. Common health risks identified across areas were illegal shebeens (drinking houses), drug abuse, illegal waste dumping and dangerous road intersections. In some areas a lack of services for the elderly was expressed and in others gender-based violence. Facility and subdistrict managers felt that this was an invaluable exercise in helping them to look beyond the walls of their facilities to understand better the needs of the communities they served.	

Overall, the research found that the values supporting the use of formal information from audits and the HIS championed rational data-driven decision-making and normative approaches to management, building vertical accountability between levels of the health system. In contrast the values underpinning the generation of local, highly-particular (perhaps informal) information supported context-specificity with a tailoring of the managerial response, and required trust within relationships. Similarly, the ability to generate experiential knowledge required an appreciation of less prescriptive approaches to management, more horizontal accountability between peers, with peers becoming accountable to each other in terms of what they shared and collectively learnt about their experience. Expressed another way, the enforcement mode was seen to value audit-type checklists and reporting of compliance, while the performance mode valued quantifiable information expressed in target-orientated indictors, and performance mode valued shared meanings and experiential knowledge. There appeared, therefore, to be a patterned connection between the governance mode and the type of information and knowledge used, which is mediated by values and the type of accountability relationships required or built.

## Discussion

The first set of findings highlighted in this study shows that there was a conflict between the nature of information valued and generated in the top-down practices of planning and management of the public health system, filtered through sub-district level practices such as monitoring progress towards service delivery targets, and the nature of information *used*, and *required* in real-world managerial decision-making at primary healthcare level. The information that was centrally valued was shown to attract management attention across the levels of the health system in terms of design, funding and implementation, as evidenced by the strong district-based HIS in this setting. In comparison, there was little support at a central level for local information. Yet local, disaggregated information and experiential knowledge are needed at facility level to make locally-appropriate and responsive decisions, and for the people management tasks required at the frontline of service delivery.

There is a well-established understanding in health information system literature that different levels of the health system have different information needs [9]. This study goes further to identify a misalignment of the nature of information needs between levels of the health systems. While existing empirical research has identified the use of ‘soft’ non-formal information in local decision-making [21–23, 53], this study begins to specify its nature, and suggests a rationale for its value. Importantly, local, disaggregated information has to be generated locally, and cannot be passed down through system planning processes. As management problems recur in local contexts, the experiential knowledge gained by learning from doing is also valuable for managers as they confront similar problems over time. This study, therefore, adds to existing literature on how managers build the expertise they need to make decisions - literature that emphasises the role of experience [54] and hence the value of reflective practice [55–57].

The second main set of findings from this study is that, despite central level influences, modes of governance operating at the subdistrict level had influence over what information was valued, generated and used locally. In this study, reflecting wider evidence [58], a central, authoritarian mode of governance, overlaid with the performance elements of a transactional mode, was found to exert influence across levels of the health system, down to the subdistrict level which had to respond to the central imperative for vertical accountability. However, the study showed that alongside a set of monitoring and supervisory practices geared towards compliance with upward reporting of performance, subdistrict managers were able to introduce some local practices which created spaces for reflective learning and co-production of new knowledge to meet local service delivery priorities. These included opportunities for problem-solving and sharing of best practice within the monthly performance management review of targets in subdistrict meetings. The different modes of governance were also linked to different sets of accountability relationships that themselves valued different types of information and knowledge. Formal routine information and checklist audits were used in vertical accountability within hierarchical relationships, while experiential knowledge was supported in peer relationships which fostered more horizontal forms of accountability.

**Fig. 4:**
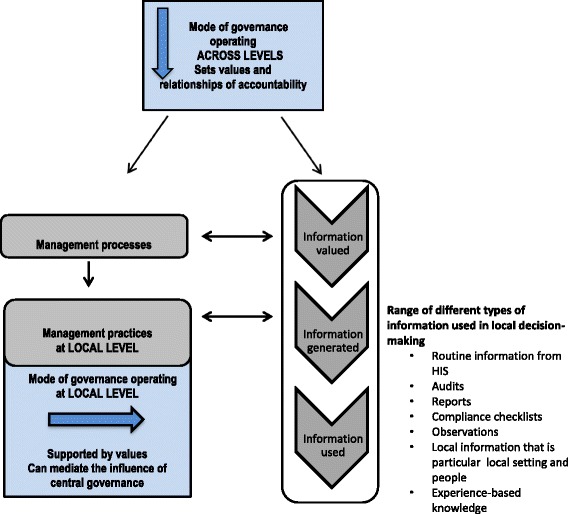
Model of how modes of governance influence information use in local decision-making

Figure 4 summarises these sets of experiences in the form of a model showing how governance processes influence the information used in local level decision-making. Although a range of information and knowledge is recognised as appropriate and desirable for decision-making at the local level of implementation, the model posits a relationship between the information that is valued and that which is generated, made available and used. Recognising governance to include responsibility for shaping how values and relationships operate to enable the health system to achieve its broader goals (see for example, [32]), the model theorises that the dominant, central mode of governance, working across levels of the health system, both places value on certain types of information and knowledge, and sets up relationships of accountability that express these values. Nonetheless, the model identifies the local level as not only responding to centrally designed management processes, but also mediating their effect through specific local-level managerial practices.

This study has several important implications for those in South Africa and other settings who are interested in strengthening local management capacity through improved use of health information. The notion of *modes of governance* makes explicit both the choices available about *how* governance is enacted and how these choices inform what information and knowledge is valued, generated and used. In most public sector bureaucracies, changing the dominant central modes of governance, which have influence across the system, is likely to be important in supporting informed local decision-making; and needs to be addressed by national policy makers and planners. However, this study has also identified a critical governance role for local-level managers who, within the framework of a district health system, are tasked with managing and supporting primary healthcare within a specified geographic area; in South Africa this is commonly termed the subdistrict level. Local level managers have a critical role in making sense of the different modes of governance - holding their respective values in a creative tension that allows both for locally responsive planning and organisational learning (horizontal forms of accountability), as well as respecting vertical forms of accountability such as upward reporting of performance. As Hill and Hupe [33] contend, modes of governance can co-exist in the same setting and each is logically equal, i.e. no one mode is better than another, as each has a particular use. The key local level managerial decision is which mode is chosen for which purpose and when, and whether the combination of modes impacting on the local level does in fact create the “structures and processes” for “solving problems and creating opportunities” - the definition of governance put forward by Kooiman [6] p69. Strengthening local level managers’ ability to create enabling environments is therefore an important leverage point in supporting informed local decision-making, and, in turn, translating national policies and priorities, including equity goals, into appropriate service delivery practices. Therefore, in securing implementation of equity goals it is not enough to establish them centrally and require compliance with related reporting needs, it is also critical to allow local level reflection on local equity-related concerns and needs to support relevant service delivery changes[59].

In addition, of particular relevance to those working in the field of health information, this study demonstrates the importance of taking an integrated, system-wide approach to supporting facility managers in using health information for decision-making. While an integrated system perspective has gained wide acceptance in overall health systems research [5, 60–62], it is less commonly used in the health information field although health information is conceptualised as a subcomponent of the health system [8]. Much work on strengthening local information use still assumes that local managers only require quantifiable routine health information, failing to acknowledge the other types of information and knowledge that managers need as well and in order to use routine health information effectively. It simply fails to acknowledge the interaction of governance and management processes and practices, as described in the model presented here, that suggests that a system-wide approach to strengthening informed decision-making is required. Yet there is a well-established body of theoretical and empirical work on the social nature of HISs [63–66] that deals with social relationships and values and that supports an integrated system view. Further attention to how different modes of governance and their values influence the generation and use of different information across the system, could provide an entry point to developing a integrated system-wide strategy for strengthening health information use in decision-making.

## Conclusion

In conclusion, this paper has offered insights into how to support the generation of the rich local information and experiential knowledge that is required by primary healthcare facility managers as a complement to information produced by formal HIS. In public health systems, recognising the influence of central modes of governance, and strengthening opportunities for stronger local governance, understood as the creation of enabling environments for local problem-solving, are important leverage points in supporting informed local decision-making.

## Abbreviations

DIALHS: District Innovation and Action Learning for Health System Strengthening
HIS: health information system
IMCI: Integrated Management of Childhood Illnesses
M&E: Monitoring and Evaluation
MDHS: Metro District Health System
PHC: Primary Health Care
WCDoH: Western Cape Department of Health

## Footnotes

1 Batho pele means ‘people first’in Sotho, one of South Africa’s official languages.
